# High-density genetic map construction and QTL mapping of a zigzag-shaped stem trait in tea plant (*Camellia sinensis*)

**DOI:** 10.1186/s12870-024-05082-9

**Published:** 2024-05-09

**Authors:** Dingding Liu, Yuanyuan Ye, Rongjin Tang, Yang Gong, Si Chen, Chenyu Zhang, Piao Mei, Jiedan Chen, Liang Chen, Chunlei Ma

**Affiliations:** Key Laboratory of Biology, Genetics and Breeding of Special Economic Animals and Plants, Ministry of Agriculture and Rural Affairs, Tea Research Institute of the Chinese Academy of Agricultural Sciences, Hangzhou, 310008 China

**Keywords:** Tea plant, Zigzag-shaped stem trait, Genetic map, QTL, Candidate genes

## Abstract

The highly unique zigzag-shaped stem phenotype in tea plants boasts significant ornamental value and is exceptionally rare. To investigate the genetic mechanism behind this trait, we developed BC_1_ artificial hybrid populations. Our genetic analysis revealed the zigzag-shaped trait as a qualitative trait. Utilizing whole-genome resequencing, we constructed a high-density genetic map from the BC_1_ population, incorporating 5,250 SNP markers across 15 linkage groups, covering 3,328.51 cM with an average marker interval distance of 0.68 cM. A quantitative trait locus (QTL) for the zigzag-shaped trait was identified on chromosome 4, within a 61.2 to 97.2 Mb range, accounting for a phenotypic variation explained (PVE) value of 13.62%. Within this QTL, six candidate genes were pinpointed. To better understand their roles, we analyzed gene expression in various tissues and individuals with erect and zigzag-shaped stems. The results implicated *CsXTH* (*CSS0035625*) and *CsCIPK14* (*CSS0044366*) as potential key contributors to the zigzag-shaped stem formation. These discoveries lay a robust foundation for future functional genetic mapping and tea plant genetic enhancement.

**Supplementary Information:**

The online version contains supplementary material available at 10.1186/s12870-024-05082-9.

## Introduction

China, the origin of tea plants (*Camellia sinensis* (L.) O. Kuntze), possesses a vast array of tea plant germplasm resources [1], including unique varieties such as albino, purple, tortuous-stem, and caffeine-free tea plants [1–4]. Current research on these specific germplasm resources predominantly focuses on leaf color and biochemical components, with minimal focus on the genetic mechanism behind the tortuous-stem trait in tea plants. This unique trait, characterized by an overall upward growth pattern and a zigzag stem shape [5], has research value, and affects the material transport and support of the above-ground part of tea plants. Despite over 3000 tea plant germplasm resources being collected and conserved in the national germplasm tea repository [1], those with zigzag-shaped stems are scarce. Among them, ‘Qiqu’, the first identified tea plant with this trait, was discovered and preserved at Wuyi Mountain by the Tea Research Institute of Fujian Academy of Agricultural Sciences in 1985. However, current research on ‘Qiqu’ remains limited, with only Cao et al. conducting a preliminary study using transcriptome sequencing and metabolome analysis [6]. Their findings suggest a possible connection between the formation of the brachytic stem in ‘Qiqu’ and gravity response and polar auxin transport. However, as the study utilized a reverse genetics approach, potential false positives may exist. Therefore, it is crucial to apply additional research strategies to corroborate these findings and investigate the underlying functional genes responsible for the zigzag-shaped stem of ‘Qiqu’.

The forward genetics, a research method employed in the study of the zigzag-shaped stem in rapeseed, has led to the identification of a critical candidate gene [7]. This approach utilizes genetic maps constructed from molecular markers and artificial hybridization populations for quantitative trait locus (QTL) mapping of significant traits [8]. When supplemented with high-quality reference genome annotation information, forward genetics facilitates the analysis of key genes regulating target traits. However, compared to annual crops like rice [9], creating an effective mapping population for tea plants suitable for constructing genetic maps and QTL mapping is challenging due to their self-incompatibility, high heterozygosity, and long growth cycle [10]. Since Grattapaglia et al proposed the “pseudo-testcross” mapping strategy [11], various molecular markers have been used to report genetic maps, including amplified fragment length polymorphisms (AFLP) [12, 13], randomly amplified polymorphic DNA (RAPD) [12, 14, 15], cleaved amplified polymorphism sequences (CAPS) [16], and simple sequence repeats (SSR) [17, 18]. Unfortunately, these linkage maps have low density, numerous gaps, and insufficient marker coverage due to limited mapping populations and molecular markers [19]. Next-generation sequencing technologies have revolutionized the genome-wide identification of single nucleotide polymorphisms (SNPs) and insertion-deletion polymorphisms in tea plant genetic resources [19]. Over the past decade, SNP markers have become instrumental in genetic mapping. Huang et al [20] utilized genotyping-by-sequencing (GBS) technology to develop 2688 SNPs, resulting in a genetic map with 15 linkage groups and an average locus spacing of 0.69 cM. Similarly, Ma et al [19] constructed a high-density linkage map with 6042 SNP markers distributed across 15 linkage groups. The recent publication of several tea reference genomes and reduced sequencing costs have led to the increased use of whole genome resequencing technology for SNP marker development in tea plants. Consequently, a high-density and high-quality genetic map with 8956 SNPs was developed from 96  F_1_ progenies of the ‘Jinxuan’ and ‘Yuncha 1’ cross [21]. These genetic maps have facilitated the identification of QTLs associated with various amino acid components, catechin components, and leaf area in tea plants. However, no studies have yet addressed the location of zigzag stem traits. This gap is attributable to the fact that all parents used for constructing genetic populations in tea plants had erect stems, rendering the resultant genetic maps unsuitable for QTL mapping of the zigzag-shaped stem trait.

This study aimed to elucidate the molecular mechanism underlying the zigzag-shaped stem formation in tea plants. We developed a BC_1_ mapping population by crossing ‘Qiqu’ with its offspring and applied whole-genome resequencing technology to analyze 58 BC_1_ offspring and their parents. Consequently, a high-density genetic map was generated, encompassing 5280 SNPs across 15 linkage groups and spanning 3328.51 cM. Furthermore, the target QTL responsible for the zigzag-shaped trait was identified on LG4. These discoveries provide valuable insights into the molecular regulation of brachytic stems, thereby broadening the potential utilization of specialized tea plant germplasm resources.

## Materials and methods

### Mapping population and DNA extraction

A cross between the‘Qiqu’ tea plant resource (male and recurrent parent) and a female parent (an offspring of ‘Qiqu’) resulted in a 58 BC_1_ mapping population. The male parent exhibited a zigzag-shaped stem, contrasting with the upright stem of the female parent. These plant materials were cultivated in the Shengzhou experimental field of the Tea Research Institute, Chinese Academy of Agricultural Sciences (CAAS), under standard horticultural conditions. DNA was extracted from the new shoots of all BC_1_ individuals and parents for subsequent experiments.

### Whole-genome sequencing of all sample

Whole-genome sequencing was employed to generate SNP markers. First, the sonication-mediated fragmentation of qualified genomic DNA was performed to create paired-end libraries, with and the End Repire Mix2 solution from the TruSeq DNA PCR-free prep kit was utilized for sequence end repair. Second, to prevent self-ligation, a base was appended to the 3’ end of each DNA sequence, followed by the addition of an adaptor with a library-specific tag to the 5’ end. This facilitated the attachment of the DNA sequence to the fell cell. Third, the BECKMAN AMPure Beads were then used to purify the library with the adaptor. Subsequent amplification of the purified DNA sequence via polymerase chain reaction (PCR) enriched the sequencing library template, which was purified again. Before sequencing, 150 insert sequences were selected, purified using 2% agarose gels, and sequenced on the Illumina HiSeq 2000 platform (Illumina, San Diego, CA, USA).

### Genotyping and construction of linkage map

Quality control and filtering of all raw reads were performed using FastQC (http://www.bioinformatics.babraham.ac.uk/projects/fastqc*)* with default parameters. The clean reads were aligned to the ‘Shuchazao’ reference genome using the Burrows-Wheeler Aligner (BWA) procedure with default parameters, generating SAM files. The mapping results were sorted in SAM tools and duplicates were marked using functions implemented in Picar [22, 23]. Uniquely mapped reads were utilized for SNP calling. SNP callings for all samples were conducted using the Haplotypecaller procedure of the GenomeAnalysis TK v4.0 software package [24], with a Fisher test of strand bias (FS) ≤ 60, Mapping quality (MQ) ≥ 40, and Quality Depth (QP) ≥ 2. High-quality SNPs were filtered using parameters such as a sequencing depth of SNP sites in two parents exceeding 5X, and a sequencing depth in the BC_1_ progeny surpassing 3X. Then, some specific types of SNP markers (ab x cd, lm x ll, nn x np, hk x hk, ef x eg) were retained based on the population type, after the filtering out of SNP markers exhibiting segregation distortion (*p *< 0.01). Linkage analysis was performed on the resultant valuable and filtered SNP markers using Onemap software [25], and ranking in each linkage group was done using the KOSAMBI method.

### Phenotype trait evaluation and QTL mapping

Between April 2020 and August 2021, observations of BC_1_ individuals’ stem morphology were conducted bimonthly. Each stem was categorized as either erect, marked ‘1’, or zigzag-shaped, marked ‘0’. Utilizing the established genetic map, zigzag-shaped trait was employed for QTL analysis. QTL IciMapping software performed the mapping via the Inclusive Composite Interval Mapping of Additive function (ICIM-ADD), with parameters set at a 1.00 cM scanning step, a *p*-value of 0.001, and a minimum 3.00 LOD threshold for significant QTLs.

### Determination of hormone content

To measure the contents of nine phytohormones, including 2-oxindole-3-acetic acid (OxIAA), indole-3-acetyl-l-aspartic acid (IAA-Asp), dihydrozeatin-O-glucoside riboside (DHZROG), N6-Benzyladenine − 9-glucoside (BAP9G), ortho-Topolin riboside (oTR), gibberellin A4 (GA4), 3-oxo-2-(2-(Z)-Pentenyl) cyclopentane-1-butyric acid (OPC-4), jasmonic Acid (JA), and jasmonoyl-L-isoleucine (JA-ILE), the node and internodes of offspring individuals with the zigzag-shaped stem, were collected as experimental materials and were immediately frozen in liquid nitrogen. These frozen samples were pulverized using a tissue lyser and extracted with a methanol/water/formic acid solution (15:4:1, v/v/v). The extraction solutions were dried via evaporation under a stream of nitrogen gas and redissolved in 100 µl methanol/ water (4:1, v/v), which were then filtered by 0.22 μm PTFE filter membrane into sample bottles, and analyzed using Ultra-performance Liquid Chromatography-Tandem Mass Spectrometry (UPLC-MS/MS). Quantitative analysis of the nine hormones in each sample was facilitated by MetWare software on the QTRAP 6500 + LC-MS/MS platform.

### Gene expression analysis of qRT-PCR

To conduct a qRT-PCR experiment for identifying candidate genes associated with the zigzag-shaped stem of tea plants, various plant parts were collected. These included the stem, apical bud, axillary bud, tender leaf, mature leaf, flower, fruit, and root of ‘Qiqu’, as well as the node and internode of offspring with erect and zigzag-shaped stems. Their total RNAs were extracted using an RNAprep Pure Plant Kit (Aidelai, Beijing, China), followed by reverse transcription using a TakaRa RR047A primeScript RT reagent kit as per the manufacturer’s instructions. Primers were designed using Primer-BLAST on the NCBI website (Table S2.). The qRT-PCR procedure was executed on a LightCycler 480 System with a LightCycler 480 SYBR Green I Master, following a previously established method [26]. Relative expression levels of the candidate genes were determined using the 2^–ΔΔCt^ method, with the *actin* gene of *CsGAPDH* serving as a internal reference standard.

## Results

### Phenotypic characterization and genetic analysis of a zigzag-shaped stem trait in tea plant

**Fig. 1:**
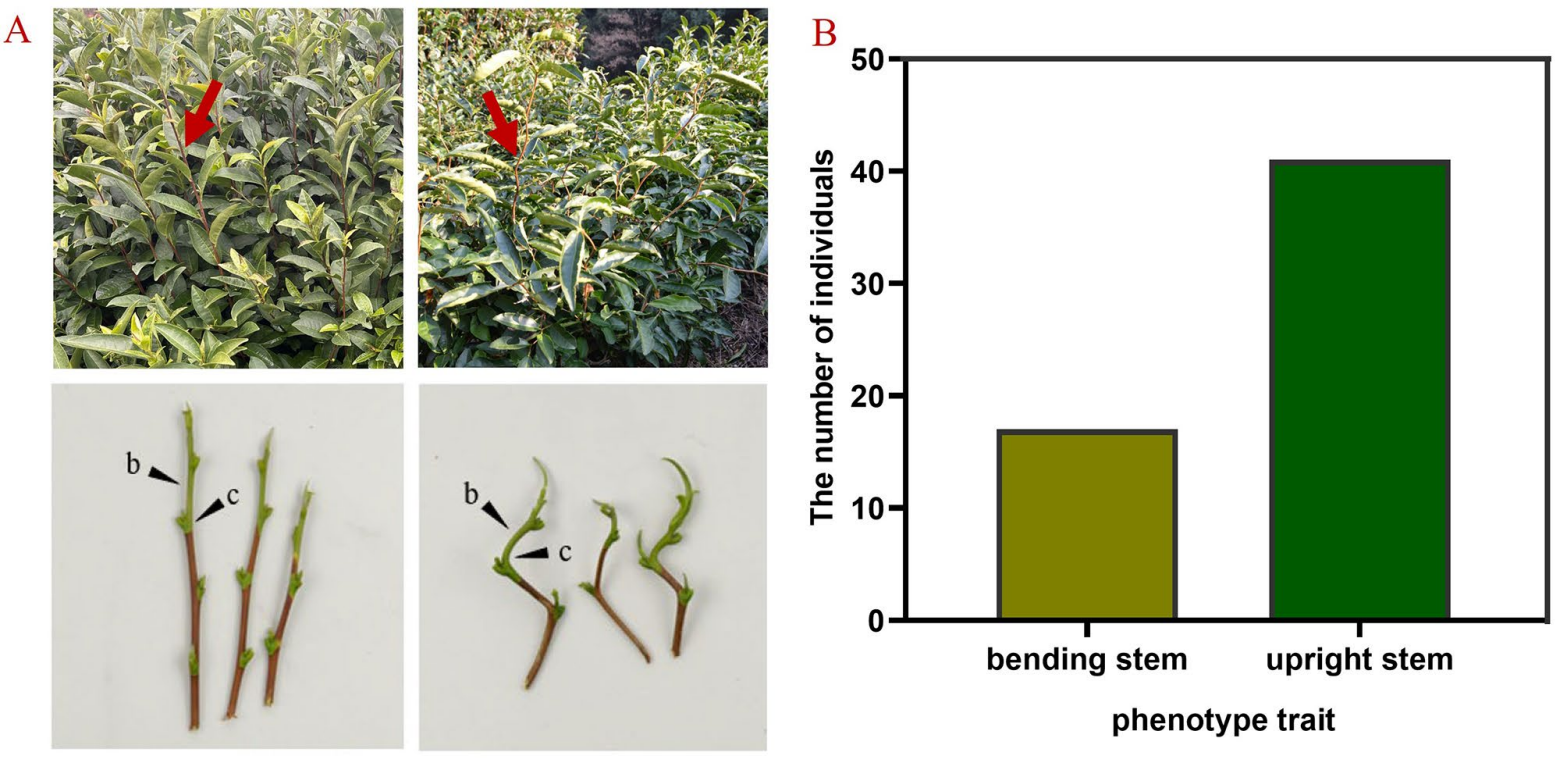
Phenotypic characterization of BC_1_ population. (**A**) Phenotype of individuals of the upright and bending stem in tea plants. (**B**) the number of individuals of upright and bending stems in tea plants

In their natural state, most tea plant stems are upright, with a few genetic resources exhibiting a zigzag-shaped stem. This trait is characterized by noticeable external bending at each leaf growing site, while the internodes maintain a typical upright position. Furthermore, we found that tea plants with this trait typically have convex leaves and wavy leaf margins (Fig. 1A). To understand the inheritance pattern of the zigzag-shaped stem, we analyzed its segregation pattern in a BC_1_ population, where the ‘Qiqu’ tea plant resource with a zigzag-shaped stem was the male, and its erect-stemmed offspring was the female. Among the 58 offspring, there was a significant difference in stem morphology: 17 had externally bent stems and 41 had upright stems. This resulted in a phenotype segregation ratio approximating 1:3 (Fig. 1B), which suggests that the zigzag-shaped stem morphology in tea plants may be a qualitative trait governed by multiple genes.

### Quality evaluation of sequencing data and SNP calling

To investigate the underlying genetic mechanisms regulating the zigzag-shaped stem in tea plants, whole-genome sequencing was performed on parents and 58 offspring using the Illumina Novaseq sequencing platform. The male and female parents yielded 205.34 and 228.38 million clean reads, respectively, achieving an average sequencing depth of ~ 8×. The offspring produced 95.40 – 138.30 million clean reads, with an average depth between 3× and 5×. In addition, over 90.58% of reads in each sample had Q30 percentages, and the GC content spanned 39.13 – 44.33%, thus confirming high sequencing data quality. Subsequently, high-quality clean reads were aligned to the ‘Shuchazao’ reference genome. This resulted in 194.86 and 217.20 million aligned clean reads for the male and female parents, with mapping rates of 98.92% and 99.10%, respectively. Among the 58 offspring, 89.68 – 130.78 million clean reads aligned, with an average mapping rate of 98.61%. Moreover, the 1× coverage reads for each sample represented 63.93 – 85.31% of the reference genome (Table S1). The SNP variation information in each sample was further analyzed. Based on the Fisher test, mapping quality, and variation reliability, a combined 104,884,365 polymorphic SNPs were identified across all samples. This included 23,262,862 heterozygous and 11,770,370 homozygous SNP sites, while the genotypes of the remaining 69,852,133 SNPs were uncertain (Table 1). These heterozygous and homozygous SNPs serve as the foundation for constructing a genetic map.

**Table 1:** Statistical summarization of the linkage map for the tea plant mapping population

Linkage Group	Chromosome	Number of Markers	Genetic Length (cM)	Physical Length (Mb)	Average Distance (cM)	Recombination Rate (cM/Mb)
LG1	Chr1	423	221.69	218.17	0.52	1.02
LG2	Chr2	337	209.14	210.79	0.62	0.99
LG3	Chr3	348	185.42	185.78	0.53	1.00
LG4	Chr4	540	299.48	186.13	0.55	1.61
LG5	Chr5	381	296.04	172.67	0.78	1.54
LG6	Chr6	355	254.67	178.98	0.72	1.42
LG7	Chr7	754	389	186.38	0.52	2.09
LG8	Chr8	386	236.44	162.73	0.61	1.45
LG9	Chr9	114	108.86	156.60	0.95	0.70
LG10	Chr10	285	189.43	165.06	0.66	1.15
LG11	Chr11	418	260.71	122.75	0.62	2.12
LG12	Chr12	178	156.06	160.43	0.88	0.97
LG13	Chr13	244	182.03	120.78	0.75	1.51
LG14	Chr14	398	248.61	133.21	0.62	1.87
LG15	Chr15	89	90.93	112.55	1.02	0.81
Average	/	350	221.90	164.87	0.69	1.35
Total	/	5250	3328.51	2473.01	/	/

### High-density genetic map construction and characteristics analysis

**Fig. 2:**
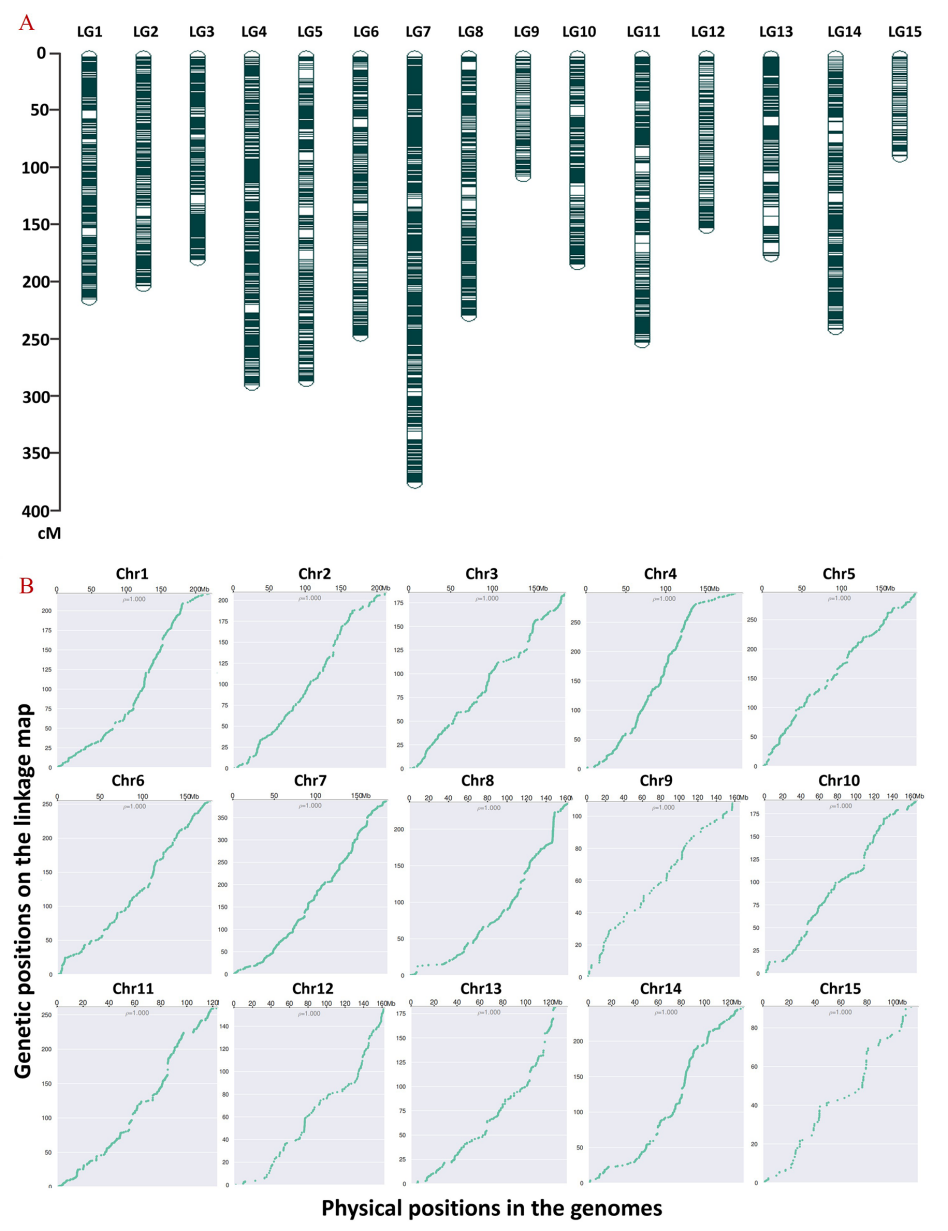
High-density linkage map constructed using genotypic data. (**A**) Distribution of SNP markers among the 15 linkage groups. (**B**) Collinearity analysis between the tea plant genetic map and the reference genome

Prior to the genetic map construction, we applied stringent filters to the SNPs based on their missing rate, sequencing depth, population type, and segregation distortion threshold (*p* < 0.01). This process yielded 22,386 high-quality SNPs for the map construction. Subsequently, 5250 SNP markers were mapped into 15 linkage groups (LGs), aligning with the chromosome number of tea plants (Fig. 2A). The total map length, as revealed by linkage analysis, was 3328.51 cM, with individual linkage group lengths ranging from 90.93 cM to 299.48 cM. LG15 was the shortest, and LG4 was the longest. On average, each linkage group contained 350 markers, varying from 89 in LG15 to 754 in LG7. The average marker distance was smallest in LG1 and LG7 (0.52) and largest in LG15 (1.02). Furthermore, the genetic map’s quality was assessed by comparing it with the reference genome, revealing a high degree of collinearity (Fig. 2B).

### QTL mapping of the zigzag-shaped stem trait in tea plant

**Fig. 3:**
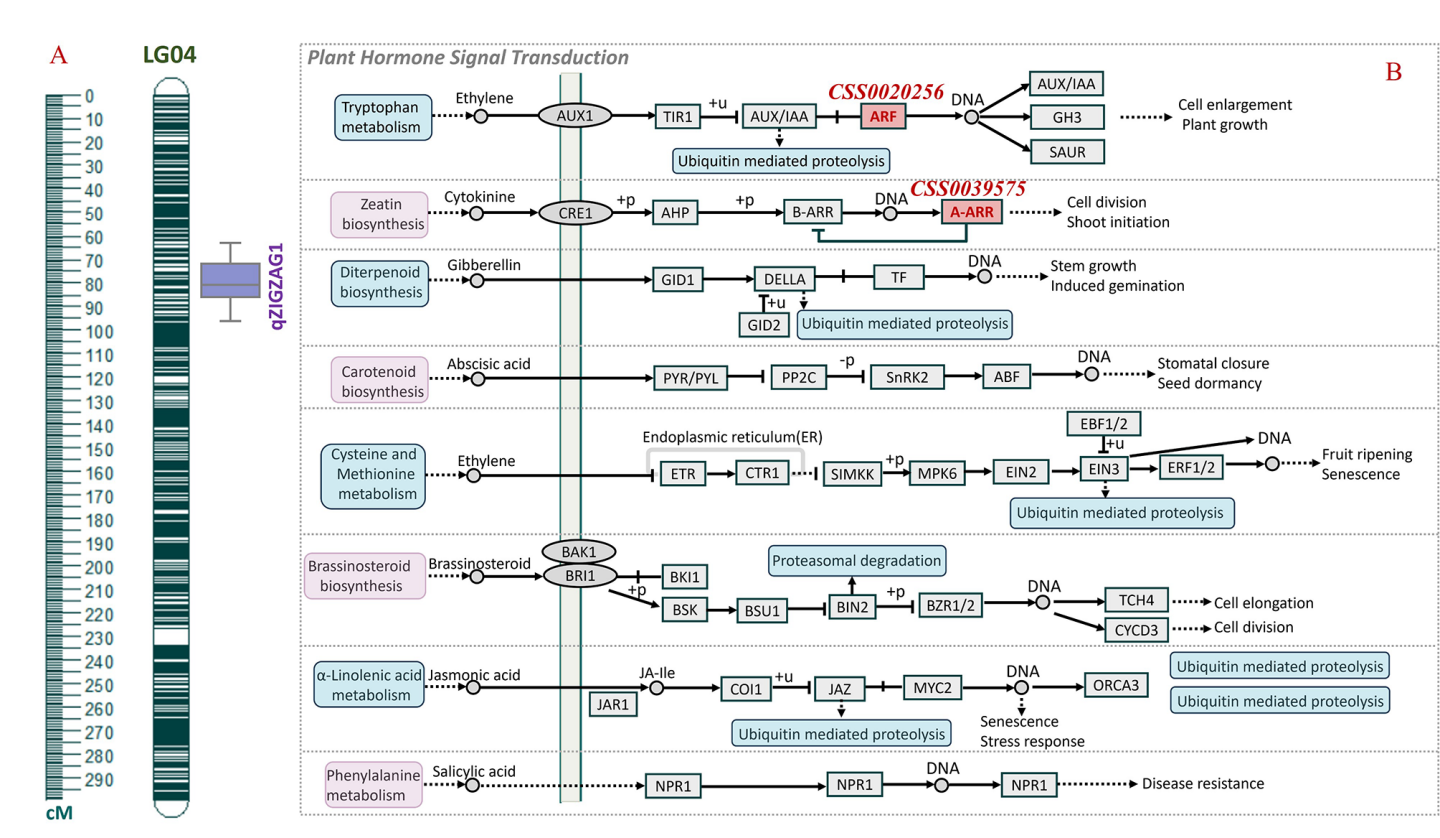
(**A**) The position of zigzag-shaped trait-related QTL in the BC_1_ population; (**B**) The location of candidate genes in plant hormone signaling pathway

Based on the constructed genetic map and phenotypic observation result of the BC_1_ population, QTL analysis was conducted for the zigzag-shaped stem trait in tea plants using the QTL IciMapping software. We considered a target QTL with an LOD score exceeding 3 as effective. This analysis led to the identification of a QTL near 122 cM of LG4, named *qZIGZAG1*. The QTL’s peak LOD score was 3.98, accounting for 13.62% of the phenotypic variance explained (PVE). Simultaneously, we identified that *qZIGZAG1* aligns with the 64.18 – 93.07 Mb region of chromosome 4, thereby deeming it a candidate genomic region (Fig. 3A).

### Prediction and analysis of candidate genes

To identify candidate genes associated with the zigzag-shaped stem formation in tea plants, we mapped candidate intervals to the ‘Shuchazao’ reference genome, and found 276 genes within these regions. Utilizing genome annotation data, we found that 137, 98, and 239 genes had functional annotations in Gene Ontology (GO), Kyoto Encyclopedia of Genes and Genomes (KEGG), and the non-redundant protein sequence database (NR), respectively. GO enrichment analysis identified gene enrichment in ‘cellular response to stimulus’, ‘signal transduction’, and ‘membrane’ categories. KEGG analysis mapped genes to ‘Photosynthesis’, ‘MAPK signaling pathway-plant’, and ‘plant hormone signal transduction’ (Fig. 3B), thereby pinpointing crucial candidate genes regulating the ‘Qiqu’ brachytic stem trait. Furthermore, zigzag-shaped stems in plants are often linked to phototropism, geotropism, and organ boundary formation, processes regulated by plant hormones and closely tied to cytoskeleton and vascular tissue development. Therefore, we preliminary screened six candidate genes within the QTL, which were xyloglucan endotransglucosylase/hydrolase (*CsXTH*: *CSS0006737*; *CSS0035625*), two-component response regulator ARR8-like (*CsARR8*: *CSS0039575*), auxin response factor 17-like (*CsARF17*: *CSS0020256*), CBL-interacting protein kinase 14 (*CsCIPK14*: *CSS0044366*), and transcription factor TCP23-like (*CsTCP23*: *CSS0010873*) (Table 2).

**Table 2:** Prediction of candidate genes underlining the QTL that related to the zigzag-shaped stem

No.	Gene_ID	Type and putative protein function	Gene_name	Physical location (bp)
1	CSS0006737	Xyloglucan endotransglucosylase/hydrolase, Putative	CsXTH	78627743_78630722
2	CSS0035625	Xyloglucan endotransglucosylase/hydrolase, Putative	CsXTH	77778550_77781514
3	CSS0039575	Two-component response regulator ARR8-like	CsARR8	64419129_64422016
4	CSS0020256	Auxin response factor 17-like	CsARF17	71883294_71884993
5	CSS0044366	CBL-interacting protein kinase 14	CsCIPK14	88391037_88394660
6	CSS0010873	Transcription factor TCP23-like	CsTCP23	92862550_92863927

### Expression pattern analysis of candidate genes

**Fig. 4:**
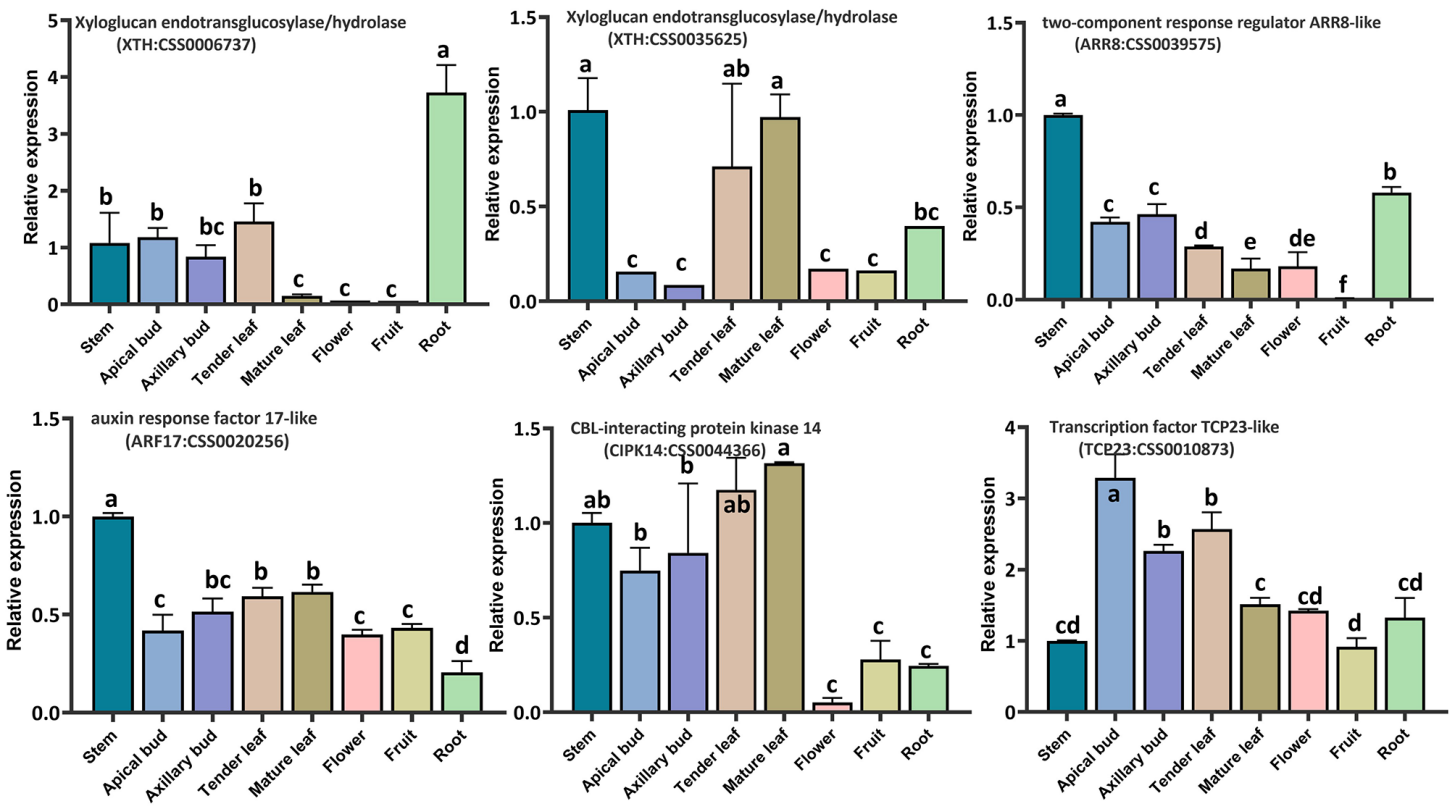
Differential expression analysis of candidate genes in different tissues of the erect individuals

**Fig. 5:**
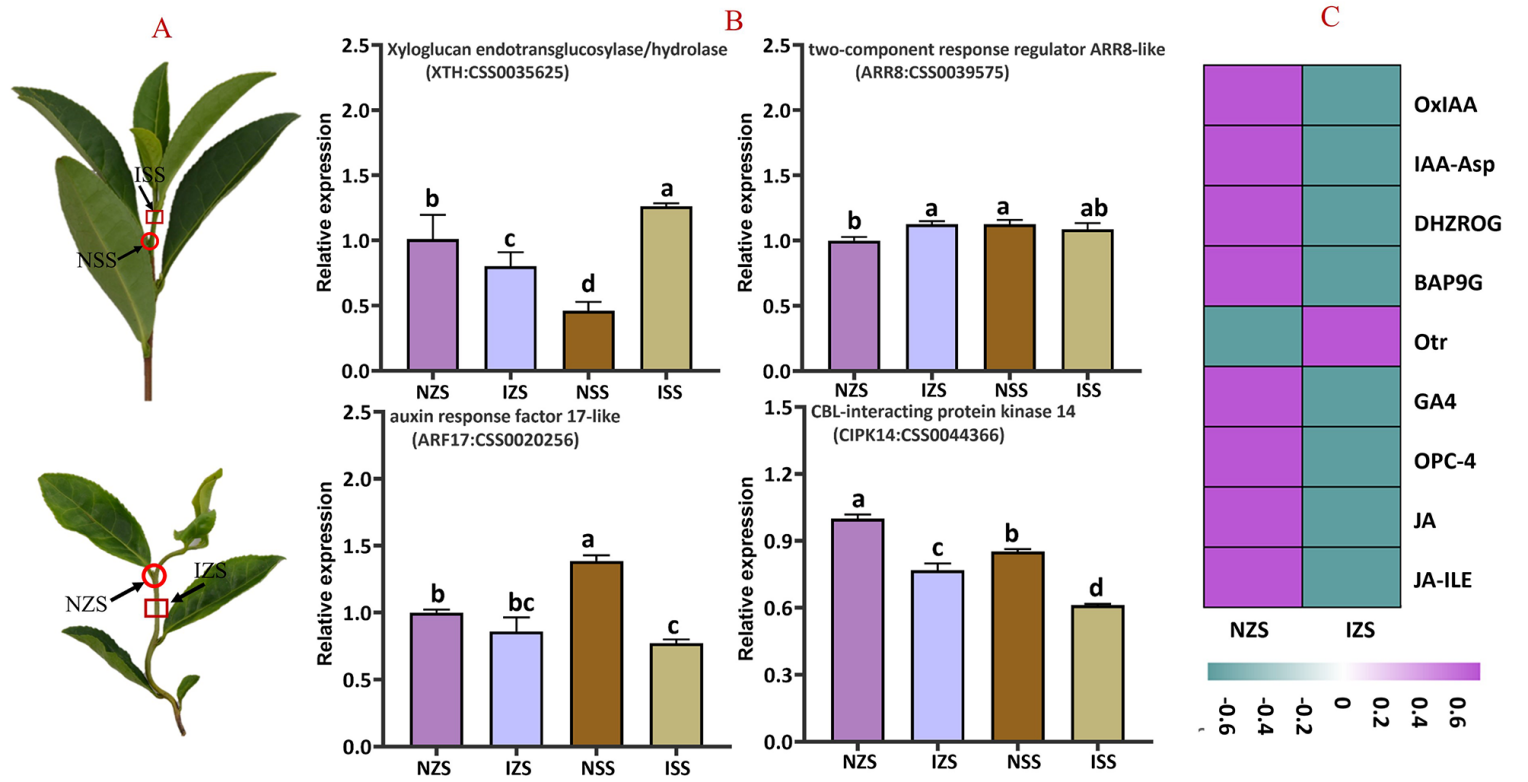
(**A**) The branch morphology of tea plants with the erect and zigzag-shaped stems. (**B**) Differential expression analysis of candidate genes in node and internode of tea plants with the straight and zigzag-shaped stem. NZS: node of zigzag-shaped stem; NSS: node of straight stem; IZS: internode of zigzag-shaped stem; ISS: internode of straight stem. (**C**) Heatmap of hormone content in the axillary bud growth point and internode sites of tea plants with the zigzag-shaped stem. OxIAA: 2-oxindole-3-acetic acid; IAA-Asp: Indole-3-acetyl-L-aspartic acid; DHZROG: Dihydrozeatin-O-glucoside riboside; BAP9G: N6-Benzyladenine − 9-glucoside; oTR: ortho-Topolin riboside; GA4: Gibberellin A4; OPC-4: 3-oxo-2-(2-(Z)-Pentenyl) cyclopentane-1-butyric acid; JA: Jasmonic Acid; JA-ILE: Jasmonoyl-L-isoleucine.

To validate the six key genes, we examined their expression patterns across eight tissues of ‘Qiqu’ using qRT-PCR, such as stem, apical bud, axillary bud, tender leaf, mature leaf, flower, fruit, and root (Fig. 4). Notably, *CSS0035625* (*CsXTH*), *CSS0039575* (*CsARR8*), *CSS0020256* (*CsARF17*), and *CSS0044366* (*CsCIPK14*) demonstrated relatively higher expression in the stem compared to other tissues. Subsequently, we conducted a detailed analysis of these genes’ expression levels in nodes and internodes of tea plants, distinguishing between straight and zigzag-shaped stems, respectively (Fig. 5A and B). *CsXTH* (*CSS0035625*) and *CsCIPK14* (*CSS0044366*) exhibited significant expression level differences between zigzag-shaped and erect individuals. Furthermore, their expression varied significantly between node and internode sites. Intriguingly, the content of two auxins, 2-oxindole-3-acetic acid and Indole-3-acetyl-L-aspartic acid, was more than twice as high in the node sites (7.14 ng/g; 5.35 ng/g) compared to the internode sites (3.38 ng/g; 2.16 ng/g) in zigzag-shaped individuals (Fig. 5C). Consequently, *CsXTH* (*CSS0035625*) and *CsCIPK14* (*CSS0044366*) emerged as key candidate genes for further investigation, aiming to illuminate the molecular mechanism underlying zigzag-shaped stem formation in tea plants.

## Discussion

Stems in higher plants originate from new shoots and are vital for plant growth and development. They serve the dual functions of transporting nutrients and providing mechanical support. Plant stems exhibit various morphological characteristics, categorized as upright, pendulous, and zigzag-shaped [5]. While most plants display upright stems certain species feature zigzag-shaped stems, including soybean [27], *Arabidopsis* [28], mulberry [29], lily [30], and rapeseed [7]. In soybeans, the brachytic stem trait’s full expression is influenced by light and temperature. Soybean plants with this trait typically exhibit lower heights, larger seeds, and more nodes, suggesting potential for increased yield under optimal conditions [31, 32]. Additionally, the genetic mechanism of the zigzag-shaped stem trait has been explored, revealing that the brachytic stem is a qualitative trait governed by one or two genes [7, 27, 33]. For example, in *Arabidopsis*, the zigzag-shaped trait at each stem node of the sgr4/zig mutation is caused by the mutation of *AtVTI11*, as revealed by mapping cloning results. Functional analysis indicates that the *AtVTI11* gene, involved in vesicle transport, decreases SNARE complex formation ability when mutated, thereby impeding auxin transport. This disruption of membrane trafficking between the trans-Golgi network and the vacuole leads to abnormalities in shoot gravitropism and morphology [28, 33–35]. Similarly, in rapeseed, a CBL-interacting protein kinase was identified as a candidate gene for the stem bend mutant, based on QTL mapping and transcriptome analysis [7]. In tea plants, a resource with a zigzag-shaped stem was also identified. To understand the molecular mechanism of the brachytic stem in ‘Qiqu’, a BC_1_ population was constructed using ‘Qiqu’ as the backcross parent. Genetic analysis indicated that the brachytic stem in tea plants is a qualitative trait controlled by a few genes.

As a third-generation molecular marker method, SNP markers have demonstrated significant value in constructing genetic maps and mapping genes associated with crucial agronomic traits. Their extreme abundance in plant genomes facilitates large-scale genotyping using high-throughput sequencing strategies such as SLAF-seq (specific-locus amplified fragment sequencing), GBS-seq, and chip array. These sequencing strategies have seen successful application in diverse species, including jujube [36], pepper [37], and rapeseed [7]. However, the SNP markers derived from these strategies are limited to random or targeted genomic regions, potentially overlooking some SNP markers linked to vital agronomic traits. Whole-genome resequencing (WGRS) technology offers a solution to these constraints. This method enables SNP site detection at any genomic location and yields a significantly larger number of SNPs compared to other sequencing strategies [21, 38]. Our study employed WGRS on the BC_1_ population of ‘Qiqu’ with the Zigzag-shaped stem, yielding 23,262,862 heterozygous and 11,770,370 homozygous SNP sites. This exceeded the SNP count reported by An et al. [21], providing a robust foundation for constructing high-density and high-quality genetic maps. These maps enhance the identification of key candidate genes regulating the zigzag-shaped stem in ‘Qiqu’. From the obtained SNP sites, we selected 5,250 SNP markers to construct a linkage map. These markers distributed evenly across 15 linkage groups, aligning with the tea plant’s haploid chromosome number. The map spanned 3328.51 cM with an average marker interval of 0.62 cM, achieving a higher map density than previously reported [20, 21, 38]. Moreover, a collinearity analysis between the genetic map and genome confirmed superior collinearity, indicating the high quality of our genetic map.

QTL mapping efficiently studies traits by identifying candidate genes that regulate specific characteristics. For instance, Zhang identified genes controlling the brachytic stem of soybean on chromosome 14, between markers BARCSOYSSR_14_1408 and BARCSOYSSR_14_1421 [39]. Similarly, candidate genes for a stem-bending mutant in rapeseed were discovered on chromosome A01, between markers Bn-A01-p2421445 and Bn-A01-p4230829 [7]. In our study, we located a QTL associated with the zigzag-shaped stem of the tea plant, accounting for 13.62% of the phenotypic variance explained, near 122 cM on LG4. This finding offers a crucial genetic locus for pinpointing candidate genes regulating the zigzag-shaped trait in ‘Qiqu’. Leveraging a high-quality reference genome of ‘Shuchazao’, we accurately narrowed down the physical regions and successful annotated candidate genes. Specifically, *qZIGZAG1* was mapped to chromosome 4 within the 61.18 to 93.07 Mb range, where 276 genes were annotated. Subsequently, preliminarily identified two *CsXTH* genes related to cell wall remodeling and four genes (*CsARR8*, *CsARF17*, *CsCIPK14*, *CsTCP23*) involved in hormone metabolism as potential candidates for the zigzag-shaped trait. XTHs reportedly significantly contribute to the loosening and rearrangement of cell walls, primarily by severing and reconnecting xyloglucans that link adjacent cellulose microfibrils, the primary structural constituent of a plant’s primary cell wall [40, 41]. The *XTH9* gene accumulates in the shoot apex region of Arabidopsis, with mutants exhibiting reduced *XTH9* gene expression displaying shorter internodal cell lengths [42]. Our study revealed relatively high expression of the *CsXTH* (*CSS0035625*) gene in the stem compared to other tissues. Particularly, the expression level in the internode sites of individuals with a zigzag-shaped stem (IZS) was lower than in the internode sites of individuals with an upright stem (ISS), aligning with Hyodo et al’s findings [42]. Interestingly, the expression level of *CsXTH* (*CSS0035625*) in the node points of individuals with the zigzag-shaped stem (NZS) significantly exceeded that in the node points of individuals with upright stems (NSS). This observation suggests that aberrant *CsXTH* expression may modify cell expansion and elongation in the node points, generating compressive forces that potentially lead to the formation of a zigzag-shaped stem. Notably, this cell expansion and differentiation are predominantly auxin-dependent [6, 43].

The gene *CSS0044366*, one of four genes associated with hormone response, displayed a high expression level in stems and a significant difference in expression between straight-stemmed and brachytic-stemmed individuals. This gene encodes the CBL-interacting protein kinase 14, a member of the CIPK family. These genes participate in the Ca^2+^-mediated plant signaling pathway, which is critical for plant development and stress response, including cold, drought, and ABA signal transduction, as well as auxin transport [44]. Tripathi et al. found that overexpressing *CaCIPK6* in chickpeas enhanced auxin sensitivity and basipetal auxin transport in the root [45]. Conversely, a decrease in basipetal root and acropetal shoot-to-root auxin transport was observed in the Arabidopsis mutation line with reduced *CIPK6* expression, suggesting that *CIPK* genes regulate plant development and growth by regulating polar auxin transport. Notably, we also found higher auxin content in the node point of zigzag-stemmed individuals, implying a potential role of phytohormones in the formation of zigzag-shaped stem. Therefore, we suggest that *CSS0035625* and *CSS0044366* are potential candidate genes for the zigzag-shaped stem trait in ‘Qiqu’.

## Conclusion

This study employed a forward genetics research strategy to investigate the genetic mechanism underlying the zigzag-shaped stem formation in ‘Qiqu’. Our genetic analysis indicated that this trait is qualitative and governed by multiple genes. Utilizing a high-density genetic map, constructed from the BC_1_ segregation population of ‘Qiqu’, we identified a QTL strongly associated with the zigzag-shaped stem. This QTL was located approximately at 122 cM on LG4, corresponding to the 61.18–93.07 Mb region on chromosome 4. Subsequently, six candidate genes potentially linked to this QTL were identified using annotation information from the reference genome. Furthermore, analysis of two auxins, 2-oxindole-3-acetic acid and Indole-3-acetyl-L-aspartic acid, in the node and internode of zigzag-shaped stem individuals led to the identification of two key candidate genes. This identification was based on expression data from various tissues of straight-stemmed individuals and from nodes and internodes of both straight and zigzag-stemmed tea plants. These discoveries offer significant insights into the molecular mechanisms behind brachytic stem formation in tea plants, thereby opening new avenues for the utilization of tea plant germplasm resources.

### Electronic supplementary material

Below is the link to the electronic supplementary material.

Supplementary Material 1

## Data Availability

The whole genome resequencing data of this BC_1_ population can be found in the NCBI Sequence Read Archive (SRA) under the bioproject number PRJNA1035020.
