# Supplementary material for: High-density genetic map construction and QTL mapping of a zigzag-shaped stem trait in tea plant (Camellia sinensis)
Source: BMC Plant Biol. 2024 May 9;24:382. doi: 10.1186/s12870-024-05082-9 (PMC11080114; doi:10.1186/s12870-024-05082-9)
Supplement: Supplementary file 1 — Supplementary Material 1 [file 12870_2024_5082_MOESM1_ESM.docx]

**Supplementary information**

**Table S1.** Quality of whole genome sequencing data

| **Sample ID** | **Read**  **number** | **Average**  **depth** | **Q30 %** | **Mapped**  **reads** | **Mapping**  **rate** | **Coverage≥1** |
| --- | --- | --- | --- | --- | --- | --- |
| Male | 205,348,952 | 7.77 | 92.46 | 194,857,126 | 98.92% | 88.64% |
| Female | 228,383,420 | 8.42 | 92.66 | 217,199,000 | 99.10% | 89.05% |
| THB_10 | 115,838,870 | 4.35 | 92.29 | 96,436,811 | 98.98% | 83.17% |
| THB_11 | 115,013,272 | 4.34 | 92.68 | 109,589,318 | 98.85% | 83.27% |
| THB_12 | 130,497,914 | 4.76 | 92.39 | 109,566,286 | 99.02% | 84.38% |
| THB_13 | 121,600,650 | 4.54 | 91.92 | 123,642,726 | 98.86% | 84.07% |
| THB_14 | 135,673,594 | 5.04 | 92.69 | 115,082,788 | 99.02% | 83.85% |
| THB_15 | 119,990,910 | 4.57 | 92.56 | 128,899,716 | 98.98% | 83.67% |
| THB_16 | 115,893,642 | 4.31 | 92.5 | 114,205,798 | 99.01% | 82.58% |
| THB_17 | 104,920,438 | 3.9 | 92.48 | 109,653,037 | 98.67% | 81.45% |
| THB_18 | 121,404,480 | 4.48 | 92.72 | 98,308,764 | 97.67% | 83.63% |
| THB_19 | 109,724,140 | 4.17 | 92.7 | 115,317,462 | 98.90% | 82.45% |
| THB_1 | 101,674,362 | 3.81 | 92.13 | 104,460,381 | 99.10% | 82.15% |
| THB_20 | 101,637,236 | 3.82 | 92.52 | 117,403,277 | 98.82% | 81.40% |
| THB_21 | 119,368,090 | 4.45 | 92.65 | 96,241,223 | 98.83% | 83.46% |
| THB_2 | 124,194,760 | 4.54 | 91.86 | 113,322,892 | 99.04% | 84.78% |
| THB_3 | 110,771,284 | 4.15 | 92.15 | 104,921,450 | 98.81% | 82.91% |
| THB_4 | 118,568,746 | 4.42 | 92.57 | 112,544,820 | 98.94% | 82.47% |
| THB_5 | 109,337,570 | 4.13 | 92.46 | 103,611,299 | 98.76% | 82.71% |
| THB_6 | 119,096,770 | 4.39 | 92.69 | 112,712,490 | 98.61% | 82.84% |
| THB_7 | 109,126,946 | 4.14 | 92.69 | 103,718,760 | 98.96% | 82.66% |
| THB_8 | 111,860,126 | 4.2 | 92.43 | 105,729,407 | 98.71% | 81.80% |
| THB_9 | 124,970,742 | 4.66 | 92.64 | 118,150,505 | 98.51% | 84.35% |
| TS_10 | 105,789,862 | 3.96 | 92.56 | 108,305,157 | 98.98% | 80.97% |
| TS_11 | 117,368,436 | 4.38 | 91.84 | 100,496,186 | 99.01% | 81.08% |
| TS1724_10 | 105,916,532 | 3.95 | 91.69 | 110,690,757 | 98.56% | 79.39% |
| TS1724_11 | 97,928,428 | 3.88 | 91.84 | 106,810,828 | 99.01% | 80.64% |
| TS1724_12 | 98,993,190 | 3.94 | 91.88 | 89,683,990 | 97.59% | 78.75% |
| TS1724_13 | 104,057,538 | 4.08 | 92.04 | 108,737,829 | 98.92% | 81.01% |
| TS1724_14 | 106,852,562 | 4.18 | 91.83 | 95,332,550 | 98.89% | 80.60% |
| TS1724_15 | 98,625,746 | 3.78 | 91.66 | 112,625,287 | 98.54% | 81.19% |
| TS1724_16 | 101,337,676 | 3.86 | 91.83 | 104,279,635 | 98.85% | 79.84% |
| TS1724_17 | 109,167,524 | 4.14 | 92.05 | 111,828,855 | 98.96% | 80.71% |
| TS1724_1 | 108,715,346 | 4.18 | 91.85 | 102,003,436 | 98.49% | 82.18% |
| TS1724_2 | 102,267,060 | 3.99 | 93.31 | 96,626,790 | 95.84% | 66.11% |
| TS1724_3 | 113,878,084 | 4.41 | 92.64 | 92,078,082 | 98.62% | 80.06% |
| TS1724_4 | 114,480,462 | 4.39 | 91.14 | 93,348,038 | 98.99% | 81.10% |
| TS1724_5 | 110,005,388 | 4.17 | 93.34 | 97,554,636 | 98.24% | 63.93% |
| TS1724_6 | 96,435,852 | 3.74 | 93.17 | 100,668,187 | 98.95% | 71.60% |
| TS1724_7 | 97,408,186 | 3.79 | 92.86 | 93,415,910 | 98.94% | 77.67% |
| TS1724_8 | 97,843,470 | 3.84 | 92.72 | 95,263,559 | 98.25% | 77.52% |
| TS1724_9 | 109,609,342 | 4.25 | 92.94 | 103,643,329 | 98.96% | 80.10% |
| TS_1 | 114,065,470 | 4.35 | 92.12 | 96,347,881 | 97.94% | 80.37% |
| TS_2 | 112,471,404 | 4.28 | 92.1 | 108,129,180 | 99.24% | 83.57% |
| TS_3 | 95,403,676 | 3.64 | 92.29 | 106,602,654 | 98.44% | 80.75% |
| TS_4 | 116,189,266 | 4.43 | 90.58 | 101,989,454 | 96.49% | 78.40% |
| TS_5 | 100,568,484 | 3.81 | 92.22 | 89,231,287 | 96.25% | 78.83% |
| TS_6 | 119,050,602 | 4.52 | 92.5 | 91,671,435 | 98.20% | 81.05% |
| TS_7 | 109,752,266 | 4.17 | 92.46 | 92,393,715 | 98.56% | 84.36% |
| TS_8 | 118,154,998 | 4.4 | 91.98 | 103,874,405 | 98.81% | 82.81% |
| ZL_37 | 116,186,948 | 4.35 | 92.26 | 109,835,245 | 98.86% | 82.94% |
| ZL_38 | 112,357,234 | 4.19 | 92.52 | 106,526,574 | 98.74% | 82.43% |
| ZL_39 | 126,091,364 | 4.6 | 92.32 | 119,393,240 | 98.94% | 83.71% |
| ZL_40 | 122,161,754 | 4.53 | 91.88 | 115,286,206 | 98.84% | 83.82% |
| ZL_41 | 138,302,780 | 5.1 | 92.54 | 130,781,411 | 98.69% | 85.31% |
| ZL_42 | 129,668,476 | 4.88 | 92.62 | 122,862,145 | 98.62% | 84.72% |
| ZL_43 | 126,126,012 | 4.67 | 92.53 | 119,257,464 | 98.65% | 84.27% |
| ZL_44 | 111,340,562 | 4.19 | 92.56 | 105,709,529 | 98.97% | 82.53% |
| ZL_45 | 135,923,634 | 4.98 | 92.68 | 128,839,764 | 98.82% | 84.96% |
| ZL_46 | 114,052,242 | 4.27 | 92.83 | 107,966,657 | 98.54% | 84.99% |

**Table S2.** Primers for qRT-PCR

| **Gene ID** | **F-Primer sequence (5'-3')** | **R-Primer sequence (5'-3')** |
| --- | --- | --- |
| GADPH | TTGGCATCGTTGAGGGTCT | CAGTGGGAACACGGAAAGC |
| CSS0006737 | CATCCTCTGGTCACCCAACT | TTATCTTCACACGGCCACCT |
| CSS0035625 | AGGGACTTGTCTGTGGATGG | TTATCTTCACACGGCCACCT |
| CSS0039575 | CGGGAATGAGCGGCTATGAT | ACTGAACCGGTTTGAGTAGGA |
| CSS0020256 | CCCTCCGTTTCTCTCTCCTC | CACCGCGTCTTCATCAACAT |
| CSS0044366 | ACCTGGAGCCGTACAAAGAG | AAGAATAGCCTCGCAGGTGT |
| CSS0010873 | CTGGGTCCTCCTCTCATGTC | CTTAGTGTGGCGGTCTTTGG |
